# Construction of Talent Competency Model for Senior Care Professionals in Intelligent Institutions

**DOI:** 10.3390/healthcare10050914

**Published:** 2022-05-13

**Authors:** Yu Song, Dongphil Chun, Peng Xiong, Xinyuan Wang

**Affiliations:** 1 Graduate School of Management of Technology, Pukyong National University, Busan 48547, Korea; songyu@pukyong.ac.kr (Y.S.); xp2020@pukyong.ac.kr (P.X.); wangxinyuan@pukyong.ac.kr (X.W.); 2 School of Economics and Management, Handan University, Handan 056000, China

**Keywords:** senior care institutions, intelligent senior care, senior care professionals, competency model

## Abstract

As the problem of the aging population becomes more and more serious, building an intelligent senior care service model and optimizing the senior care service industry become key to the development of the senior care service industry. The key to developing intelligent senior care services is to improve the overall senior care personnel quality and construct a competency model of intelligent institutional senior care professionals. This study used literature research and interviews to establish 31 relevant institutional senior care professional talent competency elements. We proposed six research propositions, prepared questionnaires for empirical analysis, and took caregivers of senior care institutions implementing intelligent management in some cities in Hebei Province, China as samples. This study established and validated 28 competency quality index models of senior care professionals in intelligent institutions in four dimensions: nursing knowledge, professional ability, personal quality, and professional attitude through exploratory factor analysis and confirmatory factor analysis. Based on the index system, this study suggests three aspects: improving the talent recruitment and selection mechanism, talent training and development mechanism, and assessment and incentive mechanism. The traditional talent competency model only focuses on fundamental aspects, such as competence. This study comprehensively establishes an evaluation model from four aspects, providing theoretical and practical significance for selecting and developing talents in intelligent institutions.

## 1. Introduction

According to a World Health Organization development report, the world’s senior population over the age of 60 will grow dramatically to 2.092 billion by 2050, accounting for 21.5% of the world’s total population [1]. According to China’s elderly dependency ratio, by 2050, there will be 45 senior citizens to support for every 100 staff members, which is nearly half of the population [2]. As the senior population grows, so will the need for long-term care services for seniors. With the development of an aging society, senior care services have become a new endeavor. The main component of senior care services is that caregivers provide daily care and a range of activities for the seniors. The quality of caregivers’ work competency will directly affect the quality of senior care [3]. The healthcare industry around the world is also under tremendous pressure. With the increase in chronic diseases in the senior population, the demands in the amount and level of nursing care have increased. However, a declining trend in caregivers in nursing facilities creates a severe contradiction between supply and demand [4]. That is why it is essential to study the competency qualities of caregivers.

Nowadays, there are fewer people in the household, and children are less willing to sacrifice their jobs to take care of their parents in the future [5]. This situation shows that more and more seniors tend to go to nursing homes, and the traditional methods of senior care services can no longer meet the actual needs of senior care. There are more mentally ill and physically challenged seniors in nursing homes, and caregivers need to provide mental illness care and complex physical care for these seniors [6]. This phenomenon poses a significant challenge to their care and accelerates the development of new senior care service models that combine intelligent technology with care services. The United States was the first country to research the development of competent care for seniors and pioneer intelligent technologies for seniors. It has identified and evaluated a range of intelligent technologies for helping caregivers to serve seniors [7]. Despite the increasing interest in intelligent care technologies in senior care institutions, there are many unknowns about the impact of intelligent technologies in the care setting. Due to the imperfect development of intelligent nursing technology, caregivers are also not skilled in the operation of intelligent technology, affecting the quality of nursing care services [8]. Therefore, we believe it is necessary to study the competency quality model of intelligent institutional senior care professionals to solve these problems.

Zhang and Lian (2019) [9] found that intelligent elderly care institutions have apparent shortcomings and deficiencies in organizational resources, technical personnel, and other technological support, resulting in low service responsiveness. Wang et al. (2021) [10] proposed that the overall lack of education and professional knowledge of intelligent elderly care talents in China and the loss of national and high-level talents result in China having a shortage in intelligent elderly professionals. From a practical point of view, the primary need in China is for professional caregivers who can use the technology, platform, and products of intelligent elderly care services.

In the past studies, most of the studies on competency models of institutional intelligent elderly care professionals have focused on developing core competency standards for caregivers, constructing competency models and evaluation indexes for caregivers, and studying seniors’ satisfaction with caregiver services and how to improve the quality of senior caregiver talents [9,11,12,13]. Scholars have focused on building models of caregiver competency and evaluation index models in terms of professional knowledge, professional skills, and personally possessed traits and service attitudes of caregivers [14,15]. The study of intelligent elderly care services mainly focuses on empirical analysis of the impact of intelligent elderly care services on the quality of institutional elderly care services. Some scholars look at the impact of intelligent senior care service content, such as life care, medical care, spiritual comfort, and other intelligent product applications, on service quality. Other scholars go from the differential impact of the seniors’ characteristics [16,17,18]. Due to the short development time of the intelligent senior care service model in China, there is a lack of quantitative methodological studies on the intelligent caregiver competency indicator model. There is minor research literature on the competency evaluation model of intelligent institutional senior care professionals. China lacks specific requirements on the competency qualities of intelligent institutional senior care professionals, especially the lack of studies on core qualities.

How to construct competency indicators to improve the competency quality of nursing staff, improve the quality of nursing services, and enhance seniors’ satisfaction with institutional intelligent senior care are key to promoting the development of senior care institutions and the construction of an intelligent senior care talent system. Therefore, the research contribution of this paper mainly enriches the research content and model by constructing a competency evaluation model for intelligent institutional senior care professionals from an empirical perspective. The high-quality intelligent caregiver competency indicator model provides strong talent support for developing senior care institutions and seniors’ different senior care needs.

## 2. Literature Review

McClelland (1973) [19] first introduced the concept of competency and established the competency model. Spencer and Spencer (2008) [20] used empirical analysis to identify the characteristics of the competency model, such as personal attributes, occupations, and professional competencies, which can clearly distinguish good employees from ordinary employees. In terms of the development process of competency, most of the studies in various countries have developed along the same lines, going through two stages: “definition and construction of the concept of competency” and “definition and construction of the concept of competency in different sectors” [21]. The current competency index system provides a vital reference for companies to examine their employees.

### 2.1. Competency Index Dimensions of Intelligent Institutional Senior Care Professionals

Nathan et al. (2018) [1] used focus group interviewing to identify a model that included nursing staff values and necessary core knowledge competencies. Sun (2014) [12] constructed a competency assessment model for nurses from four perspectives: personal expertise, trained job skills, interpersonal competency skills, and personal traits. Whelan (2006) [15] developed performance assessment indicators for nursing staff regarding professional skills, nursing staff attitudes, values, and responsibilities. Vaughn (2016) [22] constructed a model of competency elements for the rehabilitation of nursing staff with indicators including intervention care competencies, leadership, ability to care for patients, and ability to improve patients’ comfort. Stanyon and Goldberg (2017) [23] established competency evaluation indicators for registered nurses, including assessment and care planning, management procedures, and interventions, respectively, for the pain care of seniors, collaborative skills, and self-improvement skills. Campbell (2020) [24] used communication skills, analytical and assessment skills, nursing service skills, and management skills as performance indicators to evaluate the work of nurses. Yang et al. (2018) [25] constructed core evaluation indexes for professional qualification, service attitude, professional knowledge, and skill regarding nursing talents under the perspective of health care integration. Li (2021) [26] proposed that establishing a multidimensional hierarchical nursing talent competency model fits better. This study divides the competency model of intelligent institutional senior care professionals into four dimensions: nursing knowledge, professional ability, professional attitude, and personal quality.

### 2.2. Components of Nursing Knowledge Indicators

McClelland (1973) [19] suggested that educational qualifications and professional knowledge are the underlying factors in measuring talent competency performance indicators. Szczerbińska et al. (2012) [11] found that specialized nursing knowledge promotes talent-building. This expertise includes professional knowledge in nursing, medicine, and other aspects of nursing work and some psychology- and education-related knowledge. Groenewoud et al. (2015) [27] stated that the professional knowledge of the nursing staff is a critical component to ensuring safe and reliable treatment of patients with chronic depression entering geriatric institutions. Cao and Jiang (2009) [28] identified the scientific foundation module as an essential component when constructing the overall competency framework for nursing personnel development. It mainly refers to professional knowledge and knowledge of related disciplines, including medical, clinical, and nursing sciences, common diseases of the elderly, and knowledge related to traditional medicine. Davis et al. (2005) [29], in the proposed development of a competency model for nurse educators, pointed out that nurse educators teach nursing staff basic professional knowledge of senior care and medicine to improve their nursing staff with basic skills. Tous (2014) [30] proposed that professional doctors, caregivers with suitable expertise, and professional surgical techniques contribute to improving the perceived quality of the healthcare system for seniors. Sun and Zhang (2019) [31] proposed that intelligent senior caregivers should know about the Internet, 5G technology, and the Internet and senior care combined compared to traditional senior caregivers. In the era of rapid development of information management, mastering this knowledge also helps to improve the efficiency of caregivers. In summary, the indicators of nursing knowledge in this study consist of six indicators, which are elderly medical knowledge, elderly care knowledge, general geriatric knowledge, Internet, Internet of Things application knowledge, 5G technology, and elderly psychological knowledge.

### 2.3. Components of Professional Ability Indicators

McClelland (1973) [19] proposed that professional ability is a fundamental factor in measuring talent competency performance indicators in the competency model. Sun and Zhang (2019) [31] proposed to divide intelligent senior care services into four categories: daily life care, post-hospital rehabilitation, spiritual comfort, and emergency assistance. Zulas et al. (2012) [32] described the need for intelligent assistive technology for caregivers who wanted assistive technology in safety monitoring, information management systems, and daily cleaning care. Lee et al. (2013) [33] found, in a study of senior welfare centers in Seoul, Korea, that factors affecting the quality of services in senior care facilities included professionalism, reliability, health and cleanliness, responsiveness, and tangibility. Daniel et al. (2009) [34] proposed that safety services are crucial in intelligent senior care services. The low companionship of children and the loss of spouses significantly increase the dependency of the seniors. Drawing on Huang’s (2020) [35] “artificial intelligence + senior care” service model, the authors divided it into intelligent life care service, intelligent medical service, intelligent spiritual comfort service, and intelligent emergency service. Robson (2019) [36] analyzed intelligent nursing robots’ ethical and moral implications. Firstly, intelligent care robots cannot replace the implied relational commitment between caregivers and seniors. Secondly, intelligent robots cannot understand the actual needs of seniors. Therefore, intelligent technology can only aid caregiving and cannot replace caregivers. That is why caregivers must have intelligent psychological support skills. Hubbard et al. (2003) [37] suggested a lack of social interaction and interaction activities among seniors in institutionalized settings. Seniors most enjoy talking about how they feel about living in an institution. The establishment of such social relationships is helpful in terms of seniors’ quality of life and health. Caregivers need to have the ability to organize fellowship activities, interpersonal skills, and psychological support and communication skills with seniors.

Zhang and Lian (2019) [9] concluded that the lack of spiritual-comfort-type services in the intelligent senior care system resulted in low satisfaction among the senior citizens. The demand for emotional companionship and spiritual comfort services is especially prominent among senior citizens. The nursing institutions provide more basic services, such as daily care and primary nursing care. The development of application platforms for emotional care and value realization is still in its initial stage. Therefore, senior care workers should pay more attention to the psychological demands of the seniors, communicate and chat more with them, engage in psychological consultation and online chat, and hold more online and offline activities. Siegel (2014) [38] suggested that seniors’ mobility and sensory abilities are their most outstanding stress issues from a caregiver’s perspective. Seniors often feel no longer needed and have a decreased sense of social engagement, affecting their physical and mental health. Haeusermann (2018) [39] analyzed caregiving in dementia, where caregivers need to document interactions with their seniors due to the specificity of the condition. In addition to daily interactions with seniors, caregivers have various management tasks, including recording information and its transfer. They must have specific information management skills in order to be able to document the physical status of seniors better. Davis et al. (2005) [29] state that caregiver training programs also require caregivers to have the skills to train seniors in medical and health literacy.

In summary, the indicators of professional ability in this study consists of thirteen indicators, which are intelligent elderly service ability, intelligent life care ability, intelligent rehabilitation guidance ability, intelligent security monitoring ability, intelligent health monitoring ability, intelligent information management ability, interpersonal relationship ability, intelligent psychological support ability, intelligent emergency handling ability, holding online and offline association activities ability, intelligent online health and medical knowledge training, scientific research management ability, and literature search ability.

### 2.4. Components of Professional Attitude Indicators

McClelland (1973) [19] proposed that external conditions such as education and skills are not the best factors to measure performance in the competency model. Qualities such as self-concept at a deeper level can truly reflect performance indicators, which has substantial implications for constructing a competency evaluation model for intelligent senior care professionals in senior care organizations. Van der Dam et al. (2014) [40] proposed the inclusion of moral support in the care of institutionalized seniors so that professional caregivers are qualified to incorporate ethics into their daily care. Hall and Hoy (2012) [41] noted that helping seniors regain their dignity is the number one priority of caregivers’ care. Training for caregivers in respecting the privacy of seniors is essential. Backhaus (2009) [42] suggested that it is essential to pay attention to communication with seniors during the care process with them. Caregivers should care more about seniors, communicate more with them, and be polite when communicating. Smart et al. (2014) [43] suggested that caregivers with poor work ethic disengage from patients and lack concern for the seniors, and these attitudes of theirs can affect the quality of care. According to Zhu’s (2014) [44] research statistics, more than half of the employees in Nanjing nursing institutions have education at high school or below. Only 44% of the educated had received training before starting work, and the nursing jobs were poorly paid, intensive, and had a high turnover rate. It shows that most senior caregivers have weak professional self-awareness and the ability to improve.

Davis et al. (2005) [29] suggest that among the competencies nurses possess is forming values for professional nursing. It includes being law-abiding, caring for the senior, maintaining a sense of service, and an attitude of continuous improvement and learning. Su and Xu (2021) [45] suggest that artificial intelligence challenges some aspects of the legal system for senior care services, so caregivers should be law-abiding and respect seniors’ privacy. Furukawa and Kashiwagi (2021) [46] suggested that nurse managers focus on teamwork skills among the nursing staff. That staff development and support would help to improve their collective understanding and increase the nursing staff’s sense of honor, thus improving their care. Powers et al. (2010) [47] found that senior caregivers were dissatisfied with the level of support they received from managers and society in the nursing facility, which made them not develop a sense of honor for their work and led to their high turnover rate. Therefore, a sense of honor for the caregivers’ work is also essential. Love for the elderly, service consciousness, dedication, respect for the law, and responsibility are the requirements of the Chinese professional qualification standards for senior care workers [14]. In summary, this study’s indicators of a professional attitude consist of eight indicators: service awareness, hard-working, disciplined and law-abiding, respect for elderly privacy, equality and fraternity, dedication to work, professional self-awareness and improvement, and sense of honor.

### 2.5. Components of Personal Quality Indicators

McClelland’s (1973) [19] competency model argues that the factors that best measure individual behavior are personal traits and self-perceptions. Since the work of senior care services is different from other social services, the self qualities of senior care workers are essential. Senior caregivers’ professional ethics include law-abiding self-discipline, compassion, physical strength, patience, and psychological endurance [35]. Backhaus (2009) [42] suggested that an important factor that hinders communication between caregivers and seniors is that many caregivers talk to seniors only regularly. Caregivers have low expectations of communication with seniors. Caregivers do not have the time and patience. All of these factors prevent caregivers from truly understanding the needs of seniors and providing better services to them. Nonnis et al. (2020) [48] studied organizational citizenship behaviors affecting caregivers. They found that both social and environmental factors had a significant positive correlation with the positive performance of caregivers in the institution. Therefore, caregivers should have a certain level of psychological support and stress tolerance, which will increase caregiver satisfaction and thus better serve the seniors. Some seniors nowadays refuse to use intelligent devices, considering them “digital complexes.” Therefore, senior caregivers should popularize the “big intelligent device circle” for different groups of seniors with different characteristics [31]. Wang et al. (2021) [10], based on analyzing the needs of the seniors in care services and by investigating their daily living abilities, demonstrated that the primary living abilities of the seniors are weak in nursing institutions. They suggested providing personalized and customized care programs for the seniors according to their degree of disability and allocating health resources reasonably.

Zhao and Liu [49] suggested that, since the caregivers are primarily senior women, they have low psychological resistance, insufficient physical strength, and poor learning and acceptance of new things. Therefore, it leads to low service levels and low overall caregiver quality. Mosher-Ashley (2000) [50] analyzed the factors that affected college students’ choice of geriatric nursing jobs. The positive factors that affected their choice of nursing jobs are their passion for the job, the challenge, and the sense of job fulfillment. However, they also suggested negative job characteristics such as difficulty and frustration. Kanste (2006) [51] constructed a burnout scale for Finnish caregivers. The author found three factors that best characterize caregiver burnout: emotional breakdown, stress, and reduced personal fulfillment of caregivers. Szczerbińska et al. (2012) [11] suggested that caregivers should have some creativity and should provide different services depending on the situation of seniors. In summary, this study’s dimension of personal quality consists of eight indicators: physical strength, responsibility, ability to resist stress, psychological support, honesty and integrity, provide personalized service patience, nursing experience, and job achievement.

Because we sorted and summarized the above related literature findings, we initially compiled 35 competency elements for intelligent institutional professionals.

## 3. Research Design

### 3.1. Sampling and Data Collection

Hebei Province, China is the focus area of this study. The current aging situation in Hebei Province is dire. According to the general standard of China, Hebei Province has entered a moderately aging society, and the population shows the trend of aging, fewer children, emptiness, and the problem of the seniors is more and more serious. In 2019, the People’s Government of Hebei Province proposed vigorously developing intelligent senior care services and building a high-quality senior care talent team.

This paper firstly uses the interview method to revise the summarized competency evaluation elements. The interviewees are mainly senior nursing experts, nursing management personnel of nursing institutions, and senior full-time nursing personnel from some cities in Hebei Province. The authors used a 5-point Likert scale to score the initially identified indicators to determine initial intelligence agencies of old-age care professional competency assessment elements, test their validity and credibility, and use the questionnaire research method to determine the importance of the competency elements for jobs. The main targets of the questionnaire are part of the cities in Hebei Province to implement the intelligent management of nursing staff pension institutions. The questionnaire has two parts. The first part is the basic situation of the survey respondents. The second part investigates the importance of the competency elements of the nursing staff in the intelligent elderly care institutions in their posts. See Appendix A for the specific questionnaire.

The interview time was from 1–30 October 2021. A total of 40 experts participated in the interviews, including 15 senior nursing experts, 15 nursing management personnel in nursing institutions, and 10 senior full-time nursing personnel. The time of the questionnaire survey was from 15 November 2021 to 30 December 2021. The questionnaire survey adopts the sampling survey method and scoring competency elements using the Likert Five Scales. The researchers distributed 520 questionnaires, and 494 valid questionnaires were recovered, with an effective recovery rate of 95%.

### 3.2. Research Method

This study firstly summarizes and researches the literature related to competent institutional elderly care and initially summarizes the talent competency characteristic elements. Secondly, the interviewees score the initially summarized competency elements through the interview method. The researcher revised the competency elements based on the interview results and initially extracted the competency indicators. Finally, the researcher used the questionnaire method to select the sample data researched to conduct exploratory factor analysis of the indicators, conduct reliability and validity tests, extract the competency factor, and establish a competency evaluation model. In order to further verify the accuracy of the model, the author intends to use the surveyed samples to conduct a confirmatory factor analysis based on the structural equation model to determine the final competency evaluation model for senior nursing professionals in intelligent institutions.

### 3.3. Preliminary Extraction of Competency Indicators 

Based on the index framework initially determined in Chapter 2, the author conducted interviews with 40 people. Based on the interview results, the four characteristics of “scientific research management ability,” “literature search ability,” “nursing experience,” and “job achievement,” which appeared no more than 20 times in the returned questionnaires, were deleted. The competency evaluation factors of senior nursing staff in intelligent institutions were selected and determined based on the interview results. The average values of the 31 competency indexes retained in Table 1 are all greater than 4, which proves that the competency indexes of nursing staff in intelligent institutions obtained after verification by interviews are scientific and standardized. See Table 1.

### 3.4. Research Propositions

**Figure 1:**
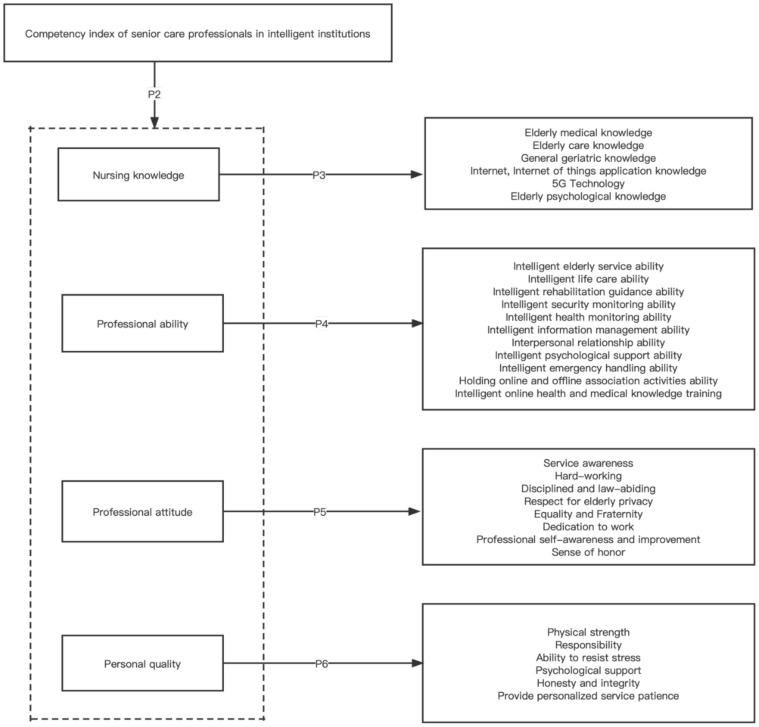
Analysis framework.

Based on the literature summary in Chapter 2 and the results of the expert interview method in Chapter 3, combined with McClelland’s (1973) [19] model of competency qualities, the authors propose the propositions of this research. The model diagram of this study appears in Figure 1.

**Proposition** **1.** *A multidimensional hierarchical model of the competency of senior care professionals in intelligent institutions has a better fit than a unidimensional structural model*.

**Proposition** **2.** *The competency model for intelligent institutional senior care professionals comprises four dimensions: nursing knowledge, professional ability, professional attitude, and personal quality*.

**Proposition** **3.** *Nursing knowledge comprises six indicators: elderly medical knowledge, elderly care knowledge, general geriatric knowledge, Internet, Internet of Things application knowledge, 5G technology, and elderly psychological knowledge*.

**Proposition** **4.** *Professional ability comprises eleven indicators: intelligent elderly service ability, intelligent life care ability, intelligent rehabilitation guidance ability, intelligent security monitoring ability, intelligent health monitoring ability, intelligent information management ability, interpersonal relationship ability, intelligent psychological support ability, intelligent emergency handling ability, holding online and offline association activities ability, intelligent online health and medical knowledge training*.

**Proposition** **5.** *Professional attitude comprises eight indicators: service awareness, hard-working, disciplined and law-abiding, respect for elderly privacy, equality and fraternity, dedication to work, professional self-awareness and improvement, and sense of honor*.

**Proposition** **6.** *Personal quality comprises six indicators: physical strength, responsibility, ability to resist stress, psychological support, honesty and integrity, provide personalized service patience*.

In order to make the article’s questionnaire and research more rigorous and accurate and express the research topic more precisely, the author defined the established indicators. See Table 2.

## 4. Data Analysis

This study mainly uses SPSS24.0 (IBM, Armonk, NY, USA) and AMOS24.0 (IBM, Armonk, NY, USA) software to conduct a statistical analysis of the surveyed data.

### 4.1. Descriptive Statistics

The survey data show that senior care nurses have the following characteristics. First, most employees are women, and the gender ratio is unbalanced. Female nurses account for 95% of the nursing staff from the questionnaire. Males account for less than 5%, and most of them are in management positions. Second, the age of the employees is old. It can be seen from the questionnaire that most of the nursing staff are over 40 years old, and less than 10% of them are under the age of 30, which hinders the construction of high-quality nursing staff. Third, nursing staff have relatively low education. About 80% of them have a junior high school degree, and only 5.47% have a college degree. The elderly care service industry requires nursing staff to have various abilities. If the education level of the nurses is low, it is not easy to provide high-quality nursing services. Fourth, job stability is poor, with 60.12% of nursing assistants working in employment positions for less than one year, which is not conducive to improving the service quality. See Table 3.

### 4.2. Data Validation

#### 4.2.1. Exploratory Factor Analysis

SPSS 24.0 was used to test the reliability and validity of the questionnaire, and the Cronbach value was 0.924, indicating that the questionnaire was stable and reliable. The KMO value is 0.899 > 0.5, indicating that the data are suitable for factor analysis. Bartlett’s sphericity test *p* = 0.000 < 0.001 indicates that the questionnaire has good construct validity and that the data are suitable for exploratory factor analysis.

Based on the data of 494 questionnaires, the principal component analysis method and the maximum rotation method are used to conduct exploratory factor analysis on 31 index items of the competency of nursing staff in intelligent institutions. There is no regulation on the number of extracted factors when extracting factors, and the characteristic value of retained factors is greater than 1. Based on the final results, we deleted factors with a common degree of less than 0.4, factors with loading coefficients greater than 0.4 on both factors, and factors with unclear explanations. For the first time, we deleted two items, “Holding online and offline association activities ability” and “Sense of honor.” We deleted “Intelligent online health and medical knowledge training” for the second time, and we deleted three items. Finally, we extracted four common factors. The results are shown in Table 4.

Among them, factor 1 is named “nursing knowledge.” Six indicators meet the requirements: elderly medical knowledge, elderly care knowledge, general geriatric knowledge, Internet, Internet of Things application knowledge, 5G technology, and elderly psychological knowledge. Factor 2 is named “professional ability.” Nine indicators meet the requirements: intelligent elderly service ability, intelligent life care ability, intelligent rehabilitation guidance ability, intelligent security monitoring ability, intelligent health monitoring ability, intelligent information management ability, interpersonal relationship ability, intelligent psychological support ability, intelligent emergency handling ability. Factor 3 is named “professional attitude.” Seven indicators meet the requirements: service awareness, hard-working, disciplined and law-abiding, respect for elderly privacy, equality and fraternity, dedication to work, professional self-awareness and improvement. Factor 4 is named “personal quality,” Six indicators meet the requirements: physical strength, responsibility, ability to resist stress, psychological support, honesty and integrity, provide personalized service patience.

#### 4.2.2. Validation Factor Analysis

The author has initially constructed a 4-dimensional competency evaluation model of intelligent institutional senior care professionals through exploratory factor analysis, but this model should be further verified. This paper uses SEM (structural equation model) to conduct validation factor analysis to verify the degree of matching between the competency index system framework established in this study and the data in the questionnaire and uses Amos24.0 to improve and verify the established model. See Table 5.

**Figure 2:**
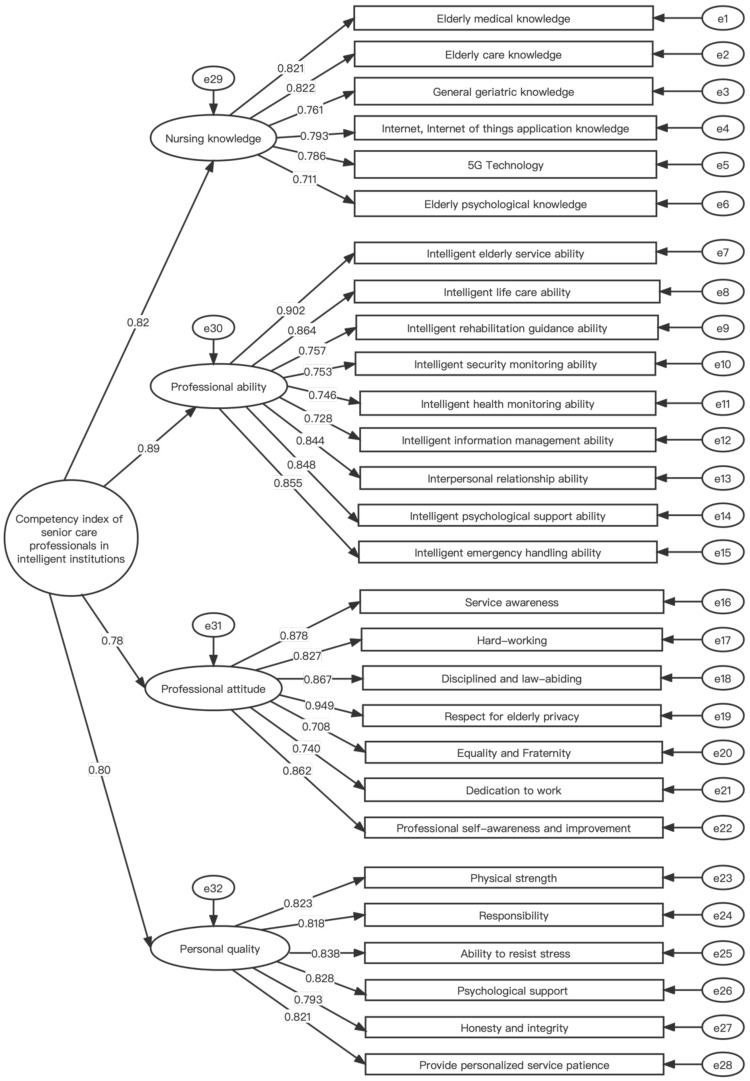
The test results of the competency index of senior care professionals in intelligent institutions.

Among them, = 2.778 < 3 indicates that the model has good structural validity. RMSEA is 0.060, less than 0.08, AGFI is greater than 0.8, NFI, IFI, CFI, and TLI values are all greater than 0.9, and GFI = 0.861, although less than 0.9, within the acceptable range. These results indicate that the established competency indicator system has an excellent fitting degree. The standardized path coefficients of the validation factor analysis of this model are in Figure 2. The factor loadings obtained from first-order factor analysis and second-order factor analysis are between 0.7 and 0.95, which can verify the rationality of the competency model established in this study.

### 4.3. Influence Effect of Various Competency Indicators of Senior Care Professionals in Intelligent Institutions

The correlation coefficient between each variable calculates the influence coefficient of each index. As shown in Equation (1), the influence effect of a particular order factor is the ratio of its factor load to a total load of all first-order factors. As shown in Equation (2), the influence effect of an observed variable on its specific order factor is its ratio of factor loading to the sum of all observed variables corresponding to a factor of an order.

As shown in Formula (3), the influence effect of an observed variable is the product of the above two ratios.

(1) $$\eta_{i}=\lambda_{i}/\sum_{i=1}^{n}\lambda_{i}$$

(2) $$\rho_{ij}=\lambda_{ij}/\sum_{j=1}^{n}\lambda_{i}$$

(3) $$\phi i_{j}=\eta_{i}\times \rho_{ij}$$

Table 6 can be obtained from the values in Figure 2.

It can be seen from Table 6 that, among the four common factors, professional ability is the most influential, followed by nursing knowledge, personal quality, and professional attitude.

Among them, the various indicators of the professional ability are, in the order of impact effect, intelligent elderly service ability, intelligent life care ability, intelligent emergency handling ability, intelligent psychological support ability, interpersonal relationship ability, intelligent rehabilitation guidance ability, intelligent security monitoring ability, intelligent health monitoring ability, and intelligent information management ability. These are the most important and could have skills for a caregiver.

According to the order of effect, the indicators are: elderly care knowledge, elderly medical knowledge, Internet, Internet of Things application knowledge, 5G technology, general geriatric knowledge, and elderly psychological knowledge. These indicators also show that the intelligent senior care combination model in the new era requires nursing staff to have basic skills and understand Internet technology and use intelligent equipment to assist the development of senior institutions better.

According to the order of influence effect, the indicators of personal quality are the ability to resist stress, psychological support, physical strength, provide personalized service patience, responsibility, honesty, and integrity. Due to the particularity of nursing service positions, nurses are under tremendous pressure, so the managers of nursing institutions should consider reducing the pressure on nursing staff to improve their work enthusiasm.

The indicators of professional attitude are respect for elderly privacy, service awareness, disciplined and law-abiding, professional self-awareness and improvement, hard-working, dedication to work, and equality and fraternity. As a senior worker, it is essential to protect the privacy of the seniors, and the intelligent senior care model requires nurses to continuously learn new knowledge and improve themselves to keep up with the pace of new innovative senior care.

## 5. Conclusions

The research object of this study is senior caregivers in senior care institutions in Hebei Province. A preliminary model of competency of senior care professionals in intelligent institutions was established based on the literature research method and interview method. The authors collected the sample data by the questionnaire method and used SPSS24.0 and AMOS24.0 software to statistically analyze the researched data, further verify and improve the competent institutional senior care professional talent competency model, and verify this research’s propositions. The specific research results are as follows:

(1) It is established that a multidimensional hierarchical model of the competency of senior care professionals in intelligent institutions has a better fit than a unidimensional structural model. The results of the validation factor analysis verified that there is a high degree of matching between each competency element. The conclusion that the competency indexes of senior care professionals in intelligent senior care institutions constructed in this study are comprehensive and systematic is the same as in the results of current mainstream studies [23,24,25,26]. When establishing the competency index system, we should comprehensively consider various factors affecting competency quality and establish multidimensional structural indicators. It is more conducive to promoting the improvement of the competency quality of talents.
(2) The competency model for intelligent institutional senior care professionals comprises four dimensions: nursing knowledge, professional ability, professional attitude, and personal quality are established. Among the four common factors, the significant effects were professional ability, nursing knowledge, personal quality, and professional attitude, in descending order. This finding is identical to the results of the current mainstream studies [12,15,21,22,23,24]. Intelligent institutional senior care professionals must be well-rounded. In addition to some fundamental indicators to measure them, some personal quality and attitudes indicators are needed to distinguish highly qualified personnel. Suppose one has good personal values and attitudes but does not have professional skills and basic knowledge. In that case, he or she will not be able to provide professional services to the seniors. Senior care is a particular job, and senior caregivers have low social status, low salary, and little room for promotion. It is not easy to become an excellent caregiver if they only have basic knowledge and skills but do not have the correct personal values and good attitude. Therefore, an excellent senior caregiver should be engaged in comprehensive development in all ability aspects.
(3) Nursing knowledge comprises six indicators: elderly medical knowledge, elderly care knowledge, general geriatric knowledge, Internet, Internet of Things application knowledge, 5G technology, and elderly psychological knowledge. This finding is identical to the results of the current mainstream studies [11,27,28,29,30,31]. The single impact effect of these six indicators was the most significant. For senior caregivers, knowledge of all aspects of care is the most basic and essential. One could have the basic knowledge to be a caregiver, and knowledge of care is equivalent to a door knocker in the talent competency model. If institutional caregivers do not have the general knowledge necessary for caregiving, they are not qualified as caregivers. Moreover, intelligent nursing is different from general nursing, and caregivers should master the knowledge of the Internet. It is essential to train caregivers’ basic knowledge. Schools and medical institutions should work together to develop a good training program and determine the program for training nursing talents. Basic nursing knowledge of caregivers is essential to improve the quality of caregivers. Since many disabled and disabled seniors are in nursing homes, caregivers should also have some general knowledge of psychology to serve these seniors better.
(4) Professional ability comprises nine indicators: intelligent elderly service ability, intelligent life care ability, intelligent rehabilitation guidance ability, intelligent security monitoring ability, intelligent health monitoring ability, intelligent information management ability, interpersonal relationship ability, intelligent psychological support ability, and intelligent emergency handling ability. These indicators are valid except for the two indicators of ability to hold online and offline association and intelligent online health and medical knowledge training, which are assumed to be invalid. This dimension has the most significant overall impact and is the most important in the competency system framework. With the basic knowledge, one could learn to apply them and can apply them in a practical context in order to further perform well as a caregiver. This conclusion is identical to most current studies [19,32,33,34,35,36,37]. In addition to nursing knowledge, professional ability is also an essential fundamental factor in measuring the performance of nursing staff. Unlike traditional care services, intelligent caregivers could have the ability to provide intelligent services and use intelligent devices to better assist them in serving the senior population. In addition to caring for the seniors, they should also have interpersonal skills to better coordinate with their families. Nowadays, most intelligent devices still exist only for primary products, so caregivers should also have the ability of psychological counseling. This finding is different from the findings of some scholars [9,29]. These scholars believe that nursing staff should have the ability to hold networking events and online lectures, but this study removed it during the exploratory factor analysis. The ability to hold events came under this study’s intelligent psychological support ability indicator. According to Davis et al. (2005) [29], the ability to organize activities and lectures are qualities that managers in nursing homes could possess, and caregivers could do their jobs. Furukawa and Kashiwagi (2021) [46] suggest that managers in nursing homes could have the ability to emphasize the organization of activities and lectures among seniors and could have the ability to organize and coordinate the relationship between caregivers and seniors.
(5) Professional attitude comprises seven indicators: service awareness, hard-working, disciplined and law-abiding, respect for elderly privacy, equality and fraternity, dedication to work, and professional self-awareness and improvement. Except for the indicator sense of honor, these indicators hold. Caregivers could have an excellent professional attitude to ensure an enthusiastic and positive approach to their work. This conclusion is identical to most current studies [41,42,43,44]. Nursing workers care for the senior population with various physical or psychological illnesses in senior care institutions, so they could have this sense of good service and love for their work to serve the senior population well. This conclusion is different from some conclusions of others [47,48], who believe that the sense of honor is also a vital component indicator. Zhao and Liu (2021) [49] believe that nurses are mostly senior women with low psychological resistance, insufficient physical strength, and poor learning and acceptance of new things. The lack of a hard-working spirit leads to a poor turnover rate.
(6) Personal quality comprises six indicators: physical strength, responsibility, ability to resist stress, psychological support, honesty and integrity, provide personalized service patience. All the indicators are valid. This finding is identical to the current mainstream research findings [11,35,51]. If each caregiver only has some essential qualities and does not have a sense of enterprise himself/herself, then the overall nursing profession will not make significant progress, and it will not be conducive to improving the quality of nursing staff competencies. Spencer and Spencer (2008) [20] suggested that these factors of personal quality are the essential indicators to differentiate talents. Due to the unique nature of nursing, caregivers should have enough physical strength to care for some special seniors. Caregivers are in a high-pressure profession. The stressful situation of caregivers is detrimental to the health of caregivers, so they should have the ability to resist stress [51]. Since seniors have different needs and different physical conditions, they could have the patience to provide them with individualized services.

Based on the study’s findings, the authors make some management recommendations. First: apply the established competency model to the recruitment of senior caregivers in senior care institutions, such as resume screening, knowledge assessment, behavioral interview, and selection and appointment. In the context of the intelligent senior care model, the professional recruitment team of senior care institutions should scientifically and systematically review the competency requirements of the required nursing staff according to the requirements of senior care institutions. Managers should develop job descriptions and recruitment plans based on the competency characteristics model. Second: managers could develop training programs and courses according to the characteristics of each employee. Universities and senior care institutions could cooperate in carrying out collaborative training for professional nursing talents required by competent institutions for senior care. By establishing a collaborative training platform for nursing talents of competent institutions for the elderly, the elderly institutions could significantly strengthen the cooperation with universities to train talents together. Administrators train nurses, geriatric caregivers, rehabilitation therapists, and health care workers. Enhance the value of talents in senior care institutions and build harmonious interpersonal relationships. It is conducive to forming good professional ethics and attitudes among caregivers to achieve high-quality talent training. Third: nursing institutions, in particular, should comprehensively and objectively evaluate the essential nursing knowledge, professional ability, professional attitude, and personal traits of nursing staff. Moreover, the job competency examination results should be the reference standard for the personal title and excellent job promotion. Strengthen the material incentive system for nursing staff in senior care and link the results of job competency evaluation with individual performance rewards and salary levels. Make nursing staff’s competence and quality linked to the salary level to improve performance.

This study still has certain limitations. First: the data used in this study are all from the data of elderly institutions in Hebei Province. The data of the research have certain limitations. In future research, the author should expand the sample size and scope of the research. Second: this study presents the concept and establishes a talent competency indicator model, which lacks empirical testing. The established indicator system should also be empirically validated to verify the impact of these talent competency factors on nursing staff performance.

## Figures and Tables

**Table 1:** Competency characteristic elements of nursing staff in intelligent institutions.

Competency Characteristics	Frequency	Score	
		Average Value	Standard Deviation
Intelligent life care ability	40	4.821	0.573
Elderly care knowledge	40	4.714	0.544
Elderly medical knowledge	40	4.723	0.534
Intelligent rehabilitation guidance ability	39	4.629	0.461
Intelligent emergency handling ability	38	4.636	0.513
Intelligent elderly service ability	36	4.591	0.392
Internet, Internet of Things application knowledge	36	4.543	0.618
5G technology	34	4.576	0.618
General geriatric knowledge	33	4.627	0.734
Intelligent psychological support ability	33	4.526	0.572
Psychological support	32	4.564	0.834
Intelligent security monitoring ability	30	4.352	0.658
Intelligent health monitoring ability	30	4.323	0.752
Dedication to work	30	4.358	0.733
Ability to resist stress	30	4.387	0.524
Respect for elderly privacy	30	4.353	0.672
Intelligent online health and medical knowledge training	29	4.412	0.642
Intelligent information management ability	28	4.424	0.649
Holding online and offline association activities ability	28	4.526	0.568
Physical strength	27	4.432	0.731
Interpersonal relationship ability	26	4.314	0.782
Responsibility	26	4.319	0.648
Service awareness	25	4.279	0.813
Disciplined and law-abiding	25	4.313	0.723
Provide personalized service patience	24	4.242	0.822
Hard-working	23	4.218	0.753
Equality and fraternity	23	4.226	0.692
Honesty and integrity	23	4.212	0.678
Sense of honor	23	4.201	0.663
Professional self-awareness and improvement	21	4.153	0.724
Elderly psychological knowledge	21	4.127	0.923
Scientific research management ability *	16		
Literature search ability *	14		
Nursing experience *	13		
Job achievement *	10		

Note: * indicates that the frequency of behavioral event interviews is less than 20 times.

**Table 2:** Definition of competency elements for intelligent institutional senior care professionals.

Serial Number	Competency Elements	Definition
1	Elderly medical knowledge	Caregivers master geriatrics pathology, physiology, and pathogenesis and common geriatric diseases.
2	Elderly care knowledge	Caregivers acquire knowledge of rehabilitation and regular disease care for the elderly.
3	General geriatric knowledge	Caregivers know the causes, treatment, and prevention of common geriatric diseases.
4	Internet, Internet of Things application knowledge	Caregivers are skilled in helping seniors operate various intelligent electronic devices and intelligent wearable devices.
5	5G technology	Caregivers use 5G technology to assist with intelligent products and services that seniors are willing to use in terms of innovative products.
6	Elderly psychological knowledge	Caregivers have the knowledge to determine the state of various aspects of senior psychology.
7	Intelligent elderly service ability	Caregivers can provide nursing, treatment, and care services for the seniors based on intelligent devices and intelligent senior care service platforms.
8	Intelligent life care ability	Caregivers have skills in caring for senior citizens in daily life, daily information pushing, and daily care.
9	Intelligent rehabilitation guidance ability	Caregivers have skills in essential medication, rehabilitation training and post-operative health monitoring, and rehabilitation guidance for senior citizens through intelligent terminal devices.
10	Intelligent security monitoring ability	Caregivers have the skills to use intelligent monitoring devices to monitor the daily activities of the seniors and the safety of their activities.
11	Intelligent health monitoring ability	Caregivers can use various intelligent devices to monitor senior citizens’ heart rate, blood pressure, and other vital signs indicators.
12	Intelligent information management ability	Caregivers’ skills to manage various data and information on seniors using an intelligent platform.
13	Interpersonal relationship ability	Caregiver skills to communicate with family members of senior citizens.
14	Intelligent psychological support ability	Caregivers can interact more with the seniors through intelligent devices and psychological counseling skills.
15	Intelligent emergency handling ability	Caregivers can apply the information received through the web platform, and caregivers carry out the emergency rescue service skills at home.
16	Holding online and offline association activities ability	Caregivers have skills in organizing structured online and offline activities for seniors.
17	Intelligent online health and medical knowledge training.	Caregivers have skills in online consultation response, online medical and health training
18	Service awareness	Caregivers can provide care to seniors in a warm, attentive, and proactive manner.
19	Hard-working	Caregivers can withstand the hard work of nursing and work long hours under exceptional circumstances.
20	Disciplined and law-abiding	Caregivers can strictly comply with regulations, penal laws, and professional ethics.
21	Respect for elderly privacy	Caregivers are fully aware of the need to protect the privacy of the seniors and not disclose their private information.
22	Equality and fraternity	Caregivers are caring and compassionate and treat the seniors they care for equally.
23	Dedication to work	Caregivers can provide care to seniors in a caring, respectful, and disciplined manner.
24	Professional self-awareness and improvement	Caregivers have the attitude of constantly learning about nursing and improving their cognitive skills.
25	Sense of honor	An individual perceived emotion of nobility that arises when caregivers feel integrated into the institutional care environment.
26	Physical strength	Caregivers have the physical strength to support and move the paralyzed seniors.
27	Responsibility	Caregivers can conscientiously and responsibly provide care services to seniors.
28	Ability to resist stress	Caregivers can de-stress when caring for disabled and semi-disabled seniors.
29	Psychological support	Caregivers can maintain an optimistic mindset in caring for seniors.
30	Honesty and integrity	The caregiver cares for the senior according to the way things are, without being influenced by personal interests, likes or dislikes, and keeping promises.
31	Provide personalized service patience	Caregivers patiently treat seniors with different needs and conditions with personalized service.

**Table 3:** Basic information of employees in pension institutions.

Variable	Category	Number of People (*n*)	Composition Ratio (%)
Gender	male	24	4.86
	female	470	95.14
Age (years)	<30	47	9.51
	30–40	217	43.93
	>40	230	46.56
Education level	primary school	60	12.15
	junior high school	167	33.81
	high school	240	48.58
	Junior college and above	27	5.47
Years of work (years)	≥1	197	39.88
	<1	297	60.12

**Table 4:** Eigenvalues and total variance of explained variation.

Factor		Initial Eigenvalue			Sum of Squared Rotating Loads		
		Total	Variance %	Cumulative Percentage %	Total	Variance %	Cumulative Percentage %
1	Nursing knowledge	4.971	16.034	16.034	4.588	14.799	14.799
2	Professional ability	6.494	20.950	36.984	6.131	19.777	34.576
3	Professional attitude	4.135	13.338	50.322	4.065	13.111	47.687
4	Personal quality	3.186	10.276	60.598	4.002	12.910	60.597

**Table 5:** Fitting parameters of competency evaluation index of intelligent institutional caring professionals.

Parameter Items	X^2^/df	RMSEA	GFI	AGFI	NNFI	TLI	CFI	IFI
Score	2.778	0.060	0.861	0.837	0.920	0.943	0.947	0.948

RMSEA: Root mean square error GFI: Goodness-of-fit index AGFI: Adjusted goodness-of-fit index NNFI: Non-normed fit index TLI: Tucker-Lewis Index CFI: Relative fit index IFI: Incremental Fit Index.

**Table 6:** Competency indicators and influence effects of senior nursing professionals in intelligent institutions.

First Order Factor	Observed Variable	Impact Effect
Nursing knowledge (0.249)	A1 Elderly medical knowledge (0.175)	0.0435
	A2 Elderly care knowledge (0.175)	0.0436
	A3 General geriatric knowledge (0.162)	0.0403
	A4 Internet, Internet of Things application knowledge (0.169)	0.0420
	A5 5G technology (0.167)	0.0416
	A6 Elderly psychological knowledge (0.151)	0.0377
Professional ability (0.271)	B1 Intelligent elderly service ability (0.124)	0.0335
	B2 Intelligent life care ability (0.118)	0.320
	B3 Intelligent rehabilitation guidance ability (0.104)	0.0281
	B4 Intelligent security monitoring ability (0.103)	0.0279
	B5 Intelligent health monitoring ability (0.102)	0.0277
	B6 Intelligent information management ability (0.1)	0.0270
	B7 Interpersonal relationship ability (0.116)	0.0313
	B8 Intelligent psychological support ability (0.116)	0.0315
	B9 Intelligent emergency handling ability (0.117)	0.0317
Professional attitude (0.238)	C1 Service awareness (0.151)	0.0359
	C2 Hard-working (0.142)	0.0338
	C3 Disciplined and law-abiding (0.149)	0.0354
	C4 Respect for elderly privacy (0.163)	0.0388
	C5 Equality and fraternity (0.121)	0.0289
	C6 Dedication to work (0.127)	0.0302
	C7 Professional self-awareness and improvement (0.148)	0.0352
Personal quality (0.242)	D1 Physical strength (0.167)	0.0389
	D2 Responsibility (0.166)	0.0386
	D3 Ability to resist stress (0.17)	0.0396
	D4 Psychological support (0.168)	0.0391
	D5 Honesty and integrity (0.161)	0.0375
	D6 Provide personalized service patience (0.167)	0.0388

## Data Availability

The data and models used during the study are available from the corresponding author by request.

## Appendix A

Questionnaire on competency characteristics of intelligent institutional senior care professionals.

Dear Ms./Mr.:

Hello! Thank you very much for participating in this survey. This questionnaire aims to analyze and study the competency of senior care professionals in intelligent institutions. We only used the survey results to construct a competency model of intelligent institutions for senior care professionals. We keep the returned questionnaires confidential. Please rate the importance of this competency in the performance of your duties and check the box corresponding to the number you selected. Your answer is a significant reference value for our research work. Thank you again for your understanding and cooperation! Good luck to you and your family at work!

**Part I. Basic Information**

1. Your gender:
  □ Male □ Female
2. Your age:
  □ <30 □ 30–40 □ >40
3. Your education:
  □ Primary School □ Junior High School □ High School
  □ College and above
4. Your Working years:
  □ ≥1 □ <1

**Part II. Investigating the importance of the competency elements of caregivers in intelligent senior care institutions**

The following items are descriptions of the daily work of institutional senior service professionals. Please tick the corresponding number.

1—Total disagree 2—Disagree 3—General 4—Agree 5—Total agree.

**Number**	**Title**	**Total Disagree–Total Agree**				
1	Elderly medical knowledge	1	2	3	4	5
2	Elderly care knowledge	1	2	3	4	5
3	General geriatric knowledge	1	2	3	4	5
4	Internet, Internet of Things application knowledge	1	2	3	4	5
5	5G technology	1	2	3	4	5
6	Elderly psychological knowledge	1	2	3	4	5
7	Intelligent elderly service ability	1	2	3	4	5
8	Intelligent life care ability	1	2	3	4	5
9	Intelligent rehabilitation guidance ability	1	2	3	4	5
10	Intelligent security monitoring ability	1	2	3	4	5
11	Intelligent health monitoring ability	1	2	3	4	5
12	Intelligent information management ability	1	2	3	4	5
13	Interpersonal relationship ability	1	2	3	4	5
14	Intelligent psychological support ability	1	2	3	4	5
15	Intelligent emergency handling ability	1	2	3	4	5
16	Holding online and offline association activities ability	1	2	3	4	5
17	Intelligent online health and medical knowledge training	1	2	3	4	5
18	Service awareness	1	2	3	4	5
19	Hard-working	1	2	3	4	5
20	Disciplined and law-abiding	1	2	3	4	5
21	Respect for elderly privacy	1	2	3	4	5
22	Equality and fraternity	1	2	3	4	5
23	Dedication to work	1	2	3	4	5
24	Professional self-awareness and improvement	1	2	3	4	5
25	Sense of honor	1	2	3	4	5
26	Physical strength	1	2	3	4	5
27	Responsibility	1	2	3	4	5
28	Ability to resist stress	1	2	3	4	5
29	Psychological support	1	2	3	4	5
30	Honesty and integrity	1	2	3	4	5
31	Provide personalized service patience	1	2	3	4	5
